# Skinfold calipers: which instrument to use?

**DOI:** 10.1017/jns.2023.58

**Published:** 2023-07-20

**Authors:** Joaquim H. Cintra-Andrade, Wagner L. Ripka, Steven B. Heymsfield

**Affiliations:** 1 Ceará State University, Fortaleza, CE, Brazil; 2 Federal University of Technology – Paraná, Curitiba, PR, Brazil; 3 Pennington Biomedical Research Center, Baton Rouge, LA, United States

**Keywords:** Anthropometry, Skinfold caliper, Skinfold thickness, Nutritional assessment

## Abstract

The considerable amount of original and generic types of skinfold calipers available is a source of systematic measurement error. This study is a brief report that critically examines the original and illustrated structural configuration of the three main types of skinfold calipers. For more than half a century, the Harpenden®, Lange® and Slim Guide® skinfolds calipers have been widely used in clinical and research settings. It is well established that the physical, mechanical and functional specificity of each type of skinfold caliper makes its interchangeable use impossible. Our report suggests that commercially available technical specifications are insufficient to judiciously choose a skinfold caliper. The area of the jaws, the coefficient of spring and the static and dynamic downward pressure of each type of skinfold caliper must be determined in the metrological laboratory and added to the technical user manual. Choosing a type of skinfold caliper for regular use, without conflict of commercial interest, requires a critical understanding of the physical, mechanical and functional characteristics that configure it. Therefore, a new downward static calibration test and the first eligibility flowchart for a skinfold caliper have been proposed. Finally, the information gathered in this report may be useful for manufacturers of anthropometric instruments and health professionals who use the skinfold technique as a tool for diagnosis and nutritional control.

## Introduction

A skinfold is composed of two parallel layers of skin and subcutaneous adipose tissue closely joined. In additional, skinfold thickness is the main surface anthropometric property for estimating the relative and absolute amount of the molecular or tissue component of total subcutaneous adiposity^(1)^. The theoretical foundation on the origin of skinfolds was documented in 1890. The French physician and anatomical artist *Paul Richer (1849–1933)* is appointed as the first researcher to determine the local and regional amount of subcutaneous adipose tissue, pinched with the fingers on the body surface, using some kind of rudimentary anthropological caliper and with limitations^(2,3)^.

Originally introduced in 1921, technical measurement standards and researches related to human body composition expanded from the 1950s onwards^(1,3)^. In 1953, *James Tanner (1920–2010)* described the first precision anthropometric instrument designed to directly and specifically measure the compressed thickness of subcutaneous adipose tissue plus the skin called the *skinfold caliper*^(4)^. Terms such as adipometer and plicometer are neologisms that should be avoided. The British instrument was optimised and introduced in 1955 and received the pseudonym Harpenden® in reference to the city where the study was carried out^(4,5)^. A few years later, a new and different skinfold caliper was introduced in 1961. The North American instrument was developed by *Karl Lange (1903–1973)* in the period from 1954 to 1957 and received the pseudonym Lange^®^ in its reference^(6)^. Later, in the 1980s, the first electronic skinfold caliper was introduced under the pseudonym Skindex^®^. In the same decade, skinfold calipers made of high-strength, low-cost plastic were proposed. The North American instrument under the pseudonym of Slim Guide® had a wider acceptance^(7,8)^. Recently, there was an important technological advance for the precision anthropometric instruments industry. Under the pseudonym Lipowise® and of Portuguese origin, the device was presented as the first generation of a digital and automated skinfold caliper with a stopwatch system and measurement recording integrated into a smartphone via Bluetooth connectivity^(9)^.

It is noteworthy that adherence and accuracy to an updated anthropometric measurement technical protocol and use of a carefully selected skinfold caliper are essential for the reproducibility and reliability of skinfold thickness^(1,10)^. For more than half a century, the Harpenden® (Baty International, UK), Lange® (Beta Technology, US) and Slim Guide® (Creative Health Products, US) skinfold calipers have been widely used in clinical settings. The first two cited stand out for having been used in research that developed and validated mathematical models to estimate body composition^(1,3)^. It is well established in the reference literature that the Harpenden®, Lange® and Slim Guide® skinfold calipers present important static and dynamic differences. The physical, mechanical and functional specificity inherent to each type of instrument makes its interchangeable use impossible^(7,11–13)^. Gruber *et al.*^(11)^ observed significant differences in skinfold thickness measurements in a sample of university students when comparing two original types of skinfold calipers. These authors also showed that the Harpenden® caliper can underestimate by 11⋅8 % the fat percentage values determined by the Lange® caliper for the mathematical model of prediction analysed. Similar results were identified in another comparative study between original and generic types of skinfold calipers^(12)^. However, Esparza-Ros *et al.*^(13)^ recently observed, in a sample of healthy young adults, a better measurement agreement between structurally similar skinfold calipers.

The considerable amount of original and generic types of skinfold calipers available, introduced bibliographically or commercially in the operational areas of anthropometry, is a source of systematic measurement error that causes questions among researchers and anthropometrists about which caliper to choose for use. Furthermore, seen as an important gap in the literature, there is no international consensus on physical, mechanical and functional reference standards for the construction and selection of these precision anthropometric instruments. Thus, this report critically examines the original and illustrated structural configuration of the three main types of skinfold calipers.

## Development

The discrimination of types of skinfold calipers is determined based on the physical–mechanical configuration that characterises them^(7)^. In some Latin American countries, mainly in Brazil, the measurement resolution of the indicator dial and the raw material of the rods are used as criteria to classify skinfold calipers as clinical or scientific. The lack of evidence in the literature^(3–8)^ suggests that this classification is unfounded and was introduced commercially by manufacturers as a strategy to overestimate instruments with high unit cost of industrial production, such as generics of original prototypes.

The physical, mechanical and functional specifications of the Harpenden®, Lange® and Slim Guide® skinfold calipers discussed in this report were reviewed and described based on original studies^(4–6)^ and evidence from important complementary experiments^(7,14–18)^. The digital illustrations presented below were made by the author of this report and are unique in their originality and richness of detail. Only the Slim Guide® skinfold caliper did not require partial disassembly.

### The Harpenden® skinfold caliper

**Fig. 1.**
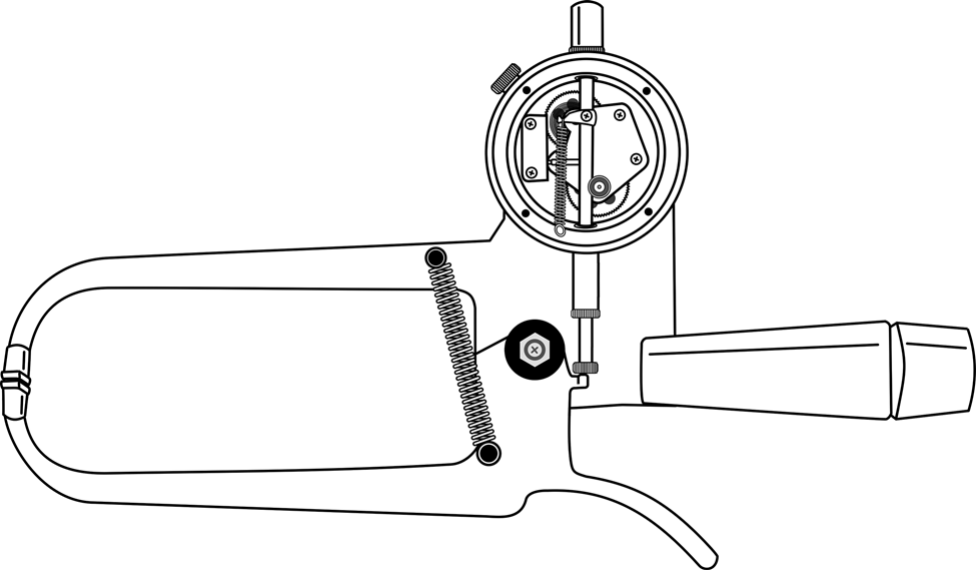
Illustrated physical–mechanical of the Harpenden^®^ skinfold caliper.

The Harpenden® skinfold caliper (Fig. 1) is made of carbon steel. The interpotent lever system is composed of pivot-spring-rod synergy. The pivot is the axis that articulates two rods 154⋅2 mm from the centre of the jaws, which are fixed and rectangular with a surface area of 90 mm² (6 × 15 mm). The non-moving upper rod supports the handle, pivot and analog dial indicator with 0⋅2 mm resolution in the range of 0 to 80 mm. The movable lower rod supports the trigger and is connected to a small cam located 15 cm from the pivot and which interacts with the plunger of the watch to determine the distance between the jaws. The two springs are installed obliquely at 16° on the lateral faces of the rods and in front of the pivot^(4,5)^. The mean caliper of static upward and downward pressures are 10⋅0 ± 0⋅25 g/mm² (900 ± 22⋅5 g) and 8⋅25 ± 0⋅25 g/mm² (742⋅5 ± 22⋅5 g), respectively, and of the mean of dynamic downward pressure are 7⋅61 ± 21 g/mm² (684⋅9 ± 18⋅9 g) and 8⋅03 ± 0⋅18 g/mm² (722⋅7 ± 16⋅2 g) at intervals of 5 and 40 mm, respectively^(14)^.

### The Lange® skinfold caliper

**Fig. 2.**
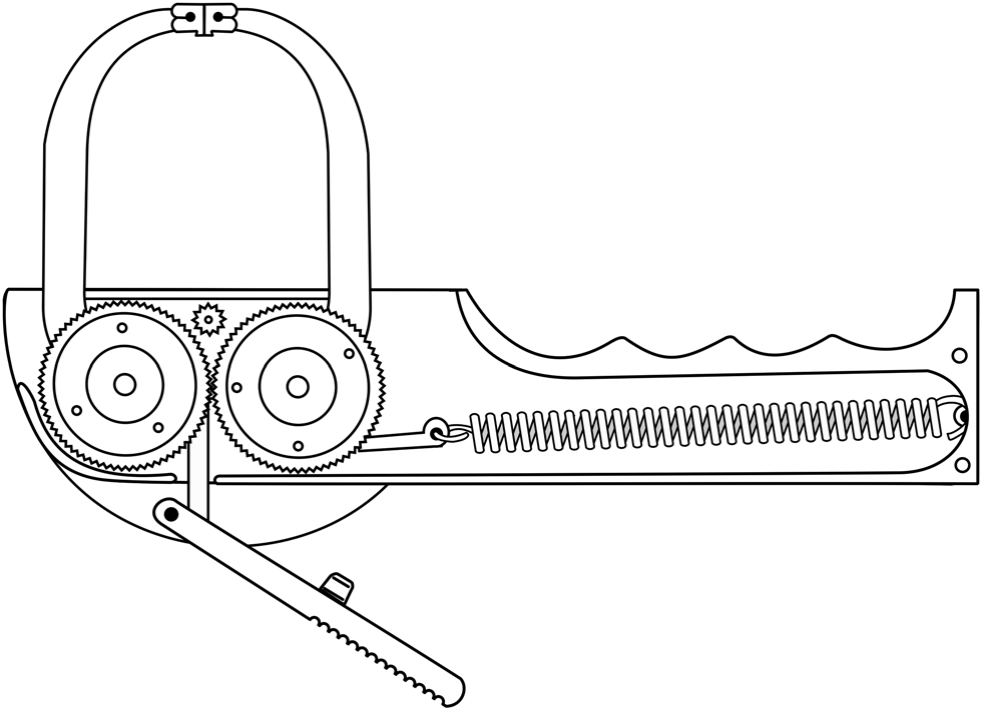
Illustrated physical–mechanical of the Lange^®^ skinfold caliper.

The Lange® skinfold caliper (Fig. 2) is compact and made of aluminium. The power transmission system is composed of spring-gear-rod synergy. A semicircular base supports the gears. The 10 mm pinion is the shaft that connects two 30 mm sprockets and interacts with the scaled indicator dial with 1⋅0 mm resolution in the range of 0 to 60 mm. It articulates two movable rods 75 mm from the centre of the jaws, which are hinged and rectangular with a surface of 30 mm² (5 × 6 mm). One rod supports and connects to the trigger and the other rod to the single spring installed transversely in the handle. The mean caliper of upward and downward static pressures are 10⋅0 ± 1⋅0 g/mm² (300 ± 30 g) and 8⋅37 ± 1⋅0 g/mm² (251 ± 30 g), respectively, at intervals of 10 to 50 mm^(6,7)^.

### The Slim Guide® skinfold caliper

**Fig. 3.**
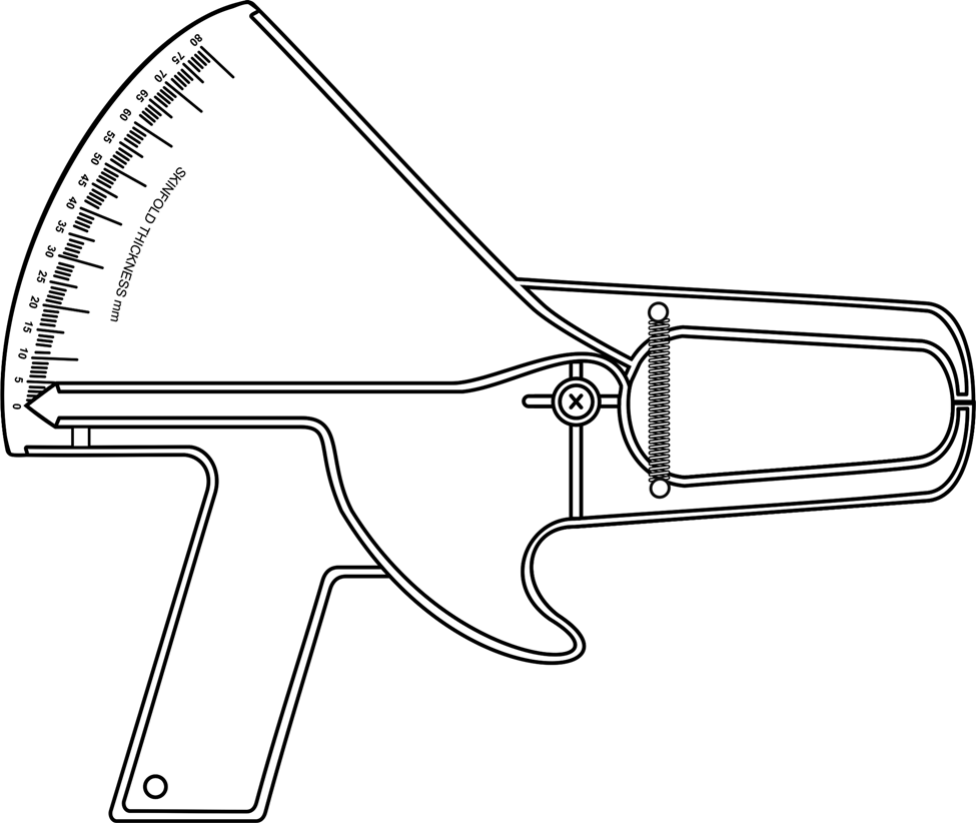
Illustrated physical–mechanical of the Slim Guide® skinfold caliper.

The Slim Guide® skinfold caliper (Fig. 3) is made from Acrylonitrile Butadiene Styrene (ABS) plastic. The interpotent lever system is composed of pivot-spring-rod synergy. The pivot is the axis that articulates two rods 110 mm from the centre of the jaws, which are fixed and rectangular with a surface area of 91 mm² (7 × 13 mm). The non-movable top rod supports the handle, pivot and scaled dial indicator with 0⋅5 mm resolution in the range of 0 to 80 mm laser engraved on a flat conical base. The movable lower rod supports the trigger and an 85 mm ferrule with an indicator. The two springs are installed vertically on the side faces of the rods and in front of the pivot. The mean caliper of upward and downward static pressures are 10⋅0 ± 1⋅0 g/mm² (910 ± 90 g) and 7⋅51 ± 1⋅0 g/mm² (683⋅4 ± 90 g), respectively, at intervals of 5 to 40 mm^(7,8)^. Dynamic downward pressure was determined but descriptive values were not presented^(15)^.

The mechanical system of interpotent lever or transmission of force inherent to each type of skinfold caliper performs static and dynamic actions with phases of upward and downward movement, corresponding to the opening and closing of the jaws or rods, respectively. The static calibration upward^(4)^ and downward^(7)^ by pressure of the jaws was determined in experiments with rudimentary methods and important limitations. The criteria employed in the studies by Edwards *et al.*^(4)^ and Schmidt and Carter^(7)^ are opposed to the dynamic downward mode of action in which the skinfold caliper is actually used to take measurements. The dynamic and static characteristics of a skinfold in response to external compression are determined by the composition, viscosity and elasticity of the skin and subcutaneous adipose tissue at each anatomical site. The phenomenon of dynamic compressibility is a morphological property easily observed in the dial indicator after applying the caliper jaws. During the first two seconds of compression, the tissue fibres are reoriented in the initial phase (0⋅0 to 0⋅5 s) followed by stretching of the elastic components (0⋅5 to 2⋅0 s) and a brief exponential decrease in the thickness skinfold. At this point, the static compressibility plateau is reached^(16,17)^. Martin *et al.*^(19)^ identified a mean of 52⋅6 % static compressibility for 13 skinfold sites using a Harpenden® skinfold caliper in a sample of male and female human cadavers. Therefore, the third second of compression is the ideal time to record a skinfold thickness measurement^(16–19)^.

Any type of skinfold caliper that is mechanically well adjusted and lubricated under normal conditions of use, in fact, exerts an upward pressure greater than the downward one, be it static or dynamic. This event is expected, because when the operator applies a manual effort to open the jaws, part of the elastic energy contained in the springs is used to overcome the frictional resistance associated with the current physical and mechanical characteristics of the moving components of the caliper. However, chronic degradation of the lubricant and microcontamination from external debris increases the coefficient of friction in the pivot, and as a result, less elastic energy will be available in the springs. Variations ≤2⋅0 g/mm² between upward and downward pressure are trivial and have historically been generalised as tolerable to any type of skinfold caliper. The mean upward pressure of 10 g/mm² produced by different instruments is the result of a variety of combinations of jaw surface area and elastic spring force that are directly proportional so that a relatively constant pressure is maintained. The Lange® skinfold caliper, which has hinged jaws, exerts low force on small surface areas, while the Harpenden® and Slim Guide® skinfold calipers, which have fixed jaws, exert large forces on large surface areas^(7)^. The spring is another physical–mechanical component that explains the variation in force between instruments. The location of attachment and the position angle of the spring affect its spring constant. Thus, the smaller the spring constant of a spring, the smaller the force required to deform it. Applying a greater force is more likely to compress the soft tissue than a smaller force. The Lange® skinfold caliper has important physical, mechanical and functional differences that make it require three times less force to open the jaws, when compared to the Harpenden® and Slim Guide® skinfold calipers^(7,13)^. Gore *et al.*^(14)^ demonstrated that, regardless of anatomical site, the skinfold thickness varies directly with spring coefficient and inversely with dynamic downward pressure. Therefore, the skinfold is underestimated or overestimated by 10 % with an increase or decrease of 1⋅0 g/mm², respectively. Another important fact to consider is that although the dial indicator has a measurement range of 60 to 80 mm, there is a safe calibration limit for the caliper mechanism to exert a downward pressure. It is noteworthy that the Harpenden® and Slim Guide skinfold calipers have a 40 mm limit while the Lange® has a 50 mm limit^(7,14,15)^.

Schmidt and Carter^(7)^ indicated that researchers and anthropometrists could be using skinfold calipers that lost their calibration and it seems that this negligence scenario remains unchanged until today. Maintenance of the pivot, springs, jaws and dial indicator of a skinfold caliper should be performed annually^(14)^. These good practices prevent the deterioration of the mechanical condition of the instrument and, consequently, maximise the reproducibility and comparability of data over a nutritional periodisation. Carlyon *et al.*^(18)^ described in detail the general calibration procedures for the components of the Harpenden® skinfold caliper. So far, only the Harpenden® and Slim Guide® skinfold calipers have been statically and dynamically calibrated^(14,15)^ in experiments where a load cell was used as a reference method^(18)^. However, despite being well defined by the authors, most calibration steps require the use of specific metrological equipment and should only be performed by professionals with proficiency in precision anthropometric instruments. Some of these procedures are reproducible in the field. Cleaning the springs regularly with a microfiber cloth and lubricating oil will prevent the chronic metal corroding effect caused by handling. The new Harpenden® skinfold caliper springs withstand up to 500 000 jaw opening and closing cycles, which is equivalent to 10⋅6 years of intensive user^(14)^. Spring coefficient is suggested as an indicator of spring replacement^(18)^.

Some generics of the Harpenden® skinfold caliper are being manufactured with a digital dial indicator and are being marketed as a technological innovation. This feature was originally experimented in 1979^(20)^ and for the current decade is considered outdated in view of the introduction of skinfold calipers with automated devices^(9)^. Although the digital dial indicator offers a faster millimeter reading, the cost benefit does not seem to be advantageous due to the fragility of this component to minimal impacts causing decalibration, thus requiring frequent repairs. Manufacturer should provide users with a way to test the performance of the caliper's downward action, a fact that seems to be ignored. The supplied steel or acrylic gauge block is restricted and limited to calibrating the dial indicator. However, they are being mistakenly used for the calibration of the pressure of the jaws, which does not apply. Recognising the limitations, foam rubber blocks are documented alternatives that have proven to be useful and reproducible for dynamic downward calibration of skinfold calipers in a clinical environment^(7)^.

**Fig. 4.**
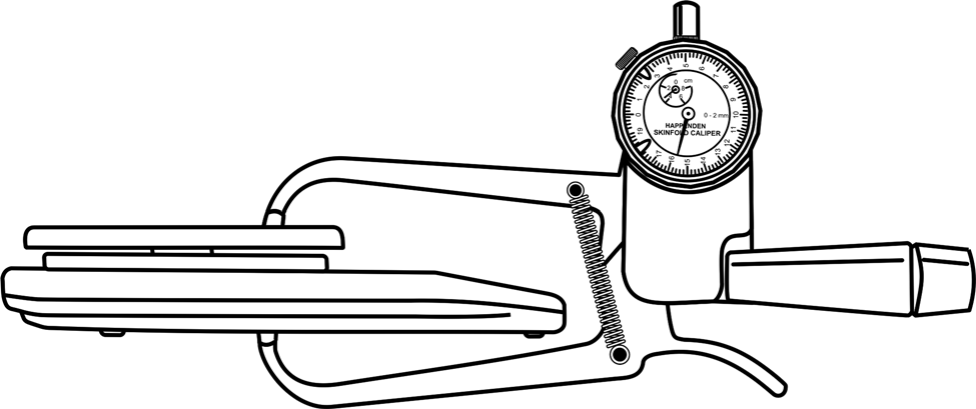
New downward static calibration test.

We also recommend a new downward static calibration test (Fig. 4) using a 0⋅1 kg precision electronic scale (Castellmaq®, PRD-0302, BR). The procedure can be applied to any type of original or generic skinfold caliper. The Harpenden® skinfold caliper was used for convenience to illustrate and describe the following test: (1) Hold the scale with your left hand. (2) Position the caliper jaws centred on the scale platform. The value determined by the scale will correspond to the elastic force of the springs for the measurement range equivalent to the known height of the scale. The model used in Fig. 4 is 38 mm and within the calibration security limit. (3) Estimate the downward static pressure (g/mm²) by dividing the spring force (g) by the caliper jaws area (mm²). The test must be performed in triplicate and the median selected. The descriptive values of downward static calibration presented in this report are suggested as a parameter. However, their use requires caution as they were determined by different methods and equipment restricted to the metrology laboratory. Thus, due to the rudimentary nature and limited measurement range, we reinforce the incentive for further studies to improve and validate the test and, finally, propose specific reference values.

**Fig. 5.**
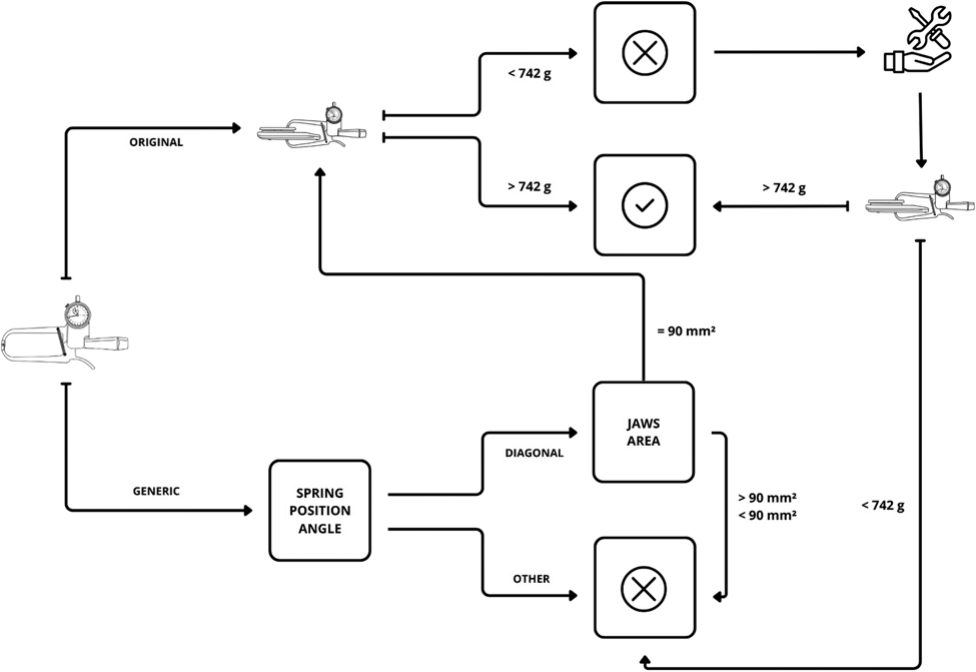
*Cintra's Flowchart*: an eligibility tool for *Type A* skinfold caliper.

Our report suggests that manufacturers can fill an important gap by providing users with an up-to-date technical data sheet on skinfold calipers. Commercially available technical specifications are limited to the resolution and measurement range, the structural dimension and a pressure value whose action and movement phase have not been defined. The isolated or combined use of this technical information is insufficient to judiciously choose a skinfold caliper. Therefore, the area of the jaws, the coefficient of spring and the static and dynamic downward pressure of each type of skinfold caliper must be determined in the metrological laboratory and added to the technical user manual. It is suggested that the three main types of skinfold calipers be categorised according to the year of bibliographic introduction: Harpenden® (*Type A*), Lange® (*Type B*) and Slim Guide® (*Type C*). Thus, the calipers developed as generics of the original prototypes could be better discriminated and chosen. Furthermore, we propose the first eligibility flowchart for an original or generic *Type A* skinfold caliper (Fig. 5). *Original skinfold caliper*: perform the downward static test (Fig. 4) to determine calibration. The 900 to 742 g range appears to be acceptable for the Harpenden® skinfold caliper. Values less than 742 g indicate that the pivot needs to be adjusted and/or the springs replaced^(18)^. Once this is done, the test should be repeated and, upon obtaining proper calibration, the caliper will be eligible for use. *Generic skinfold caliper*: a preliminary assessment of physical–mechanical components must be carried out. The spring position angle must be diagonal and the jaw area must be 90 mm²^(4,5)^. If the calipermeets these specifications, it can be subjected to downward static test to determine calibration. The value obtained will be analysed following the same procedures applied to the original skinfold caliper.

For 70 years, the recommendations by Edwards *et al.*^(4)^ for the construction of skinfold calipers were widely and internationally reproduced as a criterion. Therefore, instruments that meet these technical specifications are suggested for use by researchers and anthropometrists. A highlight is the generic version of the Harpenden® skinfold caliper manufactured by the Brazilian industry Cescorf® (Cescorf Equipment, BR). Since 1994, the performance of this *Type A* skinfold caliper of national origin has been tested in different contexts and has shown reliability in the measurements^(11,21)^. Furthermore, it was methodologically used in the development and cross-validation of mathematical prediction models^(22)^. Recently, this industry introduced the first generic *Type A* skinfold calipers manufactured with a polyacetal pivot to reduce the coefficient of friction.

## Final considerations

The information gathered in this report may be useful for manufacturers of anthropometric instruments and health professionals who use the skinfold technique as a tool for diagnosis and nutritional control. We conclude that the skinfold calipers are unique in their structural specificity and, as long as the mechanical components are pre-calibrated, any original or generic type can be used for measurements. Choosing a type of caliper for regular use, without conflict of commercial interests, requires a critical understanding of the physical, mechanical and functional characteristics that configure it. Therefore, it is absolutely necessary to regulate an international protocol of construction standards for skinfold calipers.

The Harpenden® skinfold caliper was the only one used in experiments that determined the static and dynamic calibration by pressure of the jaws and the maintenance procedures for the physical-mechanical components. In addition, it was used in *in vitro* experiments that presented descriptive values of skin thickness and compressibility of skinfolds, reproduced until today. Finally, it seems to be consolidated as the main prototype by a large part of the international academic community and by associations specialised in surface anthropometry, such as the *International Society for the Advancement of Kinanthropometry (ISAK)*. These facts do not invalidate the use of any other type of original or generic skinfold caliper mentioned or not in this study, such as the Holtain® (Holtain Ltd, UK). We project for the next decade a greater availability of structurally standardised and improved skinfold calipers with measurement automation technology and calibration methods that are more easily reproducible clinically.
